# Purification of polyphenol oxidase from tea (*Camellia sinensis*) using three-phase partitioning with a green deep eutectic solvent

**DOI:** 10.1016/j.fochx.2024.101720

**Published:** 2024-08-07

**Authors:** Yuqin Xu, Bin Zeng, Shuangling Xiao, Di Wang, Yang Liu, Shengchang Chen, Jie Teng

**Affiliations:** a Department of Tea Science, College of Agriculture, Jiangxi Agricultural University, Nanchang 330045, China; b Suichuan Tea Research Institute, Ji'an 343009, China

**Keywords:** Tea, Deep eutectic solvents, Polyphenol oxidase, Purification, Three-phase partitioning, Ammonium sulfate (PubChem CID:6097028), *t*-Butanol (PubChem CID:6386), Catechol (PubChem CID:289), Glycerol (PubChem CID:753), Menthol (PubChem CID:1254), Theaflavin (PubChem CID:4263901), Thymol (PubChem CID:6989)

## Abstract

In this study, tea polyphenol oxidase (PPO) was purified via three-phase partitioning (TPP) using a deep eutectic solvent (DES) instead of *t*-butanol. First, the properties of 13 types of synthesized DESs were characterized, and DES-7 (thymol/dodecanoic acid) was selected as the best alternative solvent. The process parameters were optimized using response surface methodology. The experimental results revealed that when the (NH_4_)_2_SO_4_ concentration, DES to crude extract ratio, extraction time, and pH were 41%, 0.5:1, 75 min, and 5.6, respectively, the recovery and purification fold of tea PPO were 78.44% and 8.26, respectively. SDS-PAGE and native-PAGE were used to analyze the PPO before and after purification of the TTP system, and the molecular weight and purification effect of PPO were detected. Moreover, the DES could be recovered and recycled. The results indicate an environmentally friendly and stable DES, and provide a reference for the large-scale application of TPP to extract PPO.

## Highlights

• DES can replace *t-*butanol in traditional three-phase partitioning (TPP) method.
• Tea PPO can be purified using DES-based TPP method.
• DES can be recycled in TPP and is environmentally friendly.
• The recovery and purification fold of PPO reached 78.44% and 8.26, respectively.

## 1 Introduction

Polyphenol oxidase (PPO) is a copper-containing terminal oxidase widely found in natural organisms that catalyzes the formation of unstable quinones using phenols. According to the different substrates of the PPO reaction, PPO is generally divided into three categories: tyrosinase (EC1.14.18.1, monophenol oxidase), catechol oxidase (EC1.10.3.1, bisphenol oxidase), and laccase (EC1.10.3.2) (Concu & Cordeiro, 2019). Catechol oxidase generally exists in plant organs and tissues and is the primary endogenous enzyme that causes the browning of fruits and vegetables (Sullivan, 2015; Tang et al., 2023). As an important enzymatic browning of transition products, quinones can be coupled with proteins, amino acid residue side chain groups, and other non-phenolic compounds, or directly with phenols to form brown polymers with higher molecular weights (Tang et al., 2023), which negatively affects the quality and shelf life of fruits and vegetables. However, PPO plays an important role in tea processing; PPO activity can be inhibited or utilized through different processing methods to produce different types of tea with differing taste and quality, such as unfermented green tea, fully fermented black tea, and slightly fermented oolong tea (Teng et al., 2021). During black tea processing, polyphenols can be oxidized to theaflavins, thearubigins, and other key components. Notably, many studies have shown the biological functions of theaflavins, including anti-inflammatory, hepatoprotective, antimutagenic, neuroprotective, and antioxidant activities (Shan, Nisar, Li, Zhang, & Wan, 2021). Owing to the roles of theaflavins' in tea quality and function, much attention has been paid to theaflavin formation from catechins in vitro via PPO from different organisms (Ding, Zou, Lu, Tong, & Chen, 2018). Some researchers have studied the functional structure and enzymatic properties of PPO from apple (Han, Liu, Hao, & Ni, 2020), tea (Teng et al., 2017), potato (Mine, 2020), borage (Alici & Arabaci, 2016), and Pacific white shrimp (Sae-Leaw, Benjakul, & Simpson, 2017), and have found that the molecular weight of PPO from different organisms is generally between 45 and 130 kDa. Further, different isoforms of PPOs may exist in one organism; this may be attributed to the wide range of PPO sources and complexity of gene expression. Therefore, the separation, purification, and crystallization of PPO are relatively difficult, and detailed information regarding its advanced structure is not yet available (Tang et al., 2023).

Extracting, separating, and purifying PPO from organisms enables the study of its enzymatic properties, enzymatic oxidation in vitro, and biotransformation. Owing to their unique physical and chemical properties, enzymes can be separated and purified using chromatographic methods, including ion exchange, gel filtration, reversed-phase, and affinity chromatography, as well as via hydrophobic interactions. The selection of the chromatographic method depends on the organism, charge, and molecular weight of the PPO, and several types of chromatography are used to obtain high-purity proteins (Teng et al., 2017). However, certain impurities are typically removed via salting-out precipitation, temperature induction, three-phase partitioning (TPP), and aqueous two-phase methods before chromatography (Alici & Arabaci, 2016). Ke et al. (2021) used ammonium sulfate precipitation, ultrafiltration membranes, and UNOsphere Q anion-exchange chromatography to purify and identify a heat-resistant glycosyl protein. This protein had a molecular weight of 66 kDa and was obtained from fresh leaves and tea soup (*Camellia sinensis* var. “Zhengshanxiaozhong”). Moreover, it exhibited PPO activity, which may be due to non-enzymatic protein glycosylation or the formation of a PPO–tea polyphenol complex in tea soup. Derardja, Pretzler, Barkat, and Rompel (2024) isolated and purified PPO via acetone powder homogenate, ammonium sulfate precipitation, Q-Sepharose fast-flow anion chromatography, and Mono S HR 5/50 GL cation-exchange chromatography from olives (*Olea europaea* L., cv. Chemlal). The PPO monomer had an enzyme specific activity, purification fold, and molecular weight of 203.9 U/mg, 17.13, and 54 kDa, respectively. The fine purification of enzymes can yield higher-purity proteins; however, expensive equipment, complicated technical requirements, complex operating specifications, and other limitations make it unsuitable for large-scale production (Hassan et al., 2024; Tahir et al., 2024; Zou, Zhang, Xu, & Yin, 2024).

TPP involves the formation of three phases upon the addition of salts, such as ammonium sulfate, and water-soluble alcohols, such as *t*-butanol, to the aqueous extract or slurry of the source. TPP contains three immiscible continuous liquid phases. Because of differences in the physicochemical properties and hydrophilicity of these three liquid phases, the distribution difference between the target and phase is formed by controlling the phase formation conditions (Kumar & Rapheal, 2011). Compared with the two-phase extraction system, TPP can quickly achieve the simultaneous separation of multiple target components, simplify the operation process, improve production efficiency, and achieve the enrichment of specific targets by artificially regulating the microstructure of different phases (Chew, Ling, & Show, 2019). Therefore, TPP can simultaneously extract and purify numerous biomolecules including proteins, active ingredients, oils, and pigments (Alici & Arabaci, 2016; Chew et al., 2019; Hu, Chen, & Tan, 2023). TPP was used to purify PPO from borage for the first time. The purification fold of PPO after separation reached 3.59, and the total enzyme activity recovery rate was 68.75% (Alici & Arabaci, 2016). Although TPP has several advantages over traditional extraction separation technology, certain aspects require further research and improvement, particularly the organic solvent *t*-butanol, which is widely used in TPP systems. However, *t*-butanol is flammable, volatile, explosive, and harmful to the environment (Chen, Cai, & Tan, 2022). Therefore, the development of a TPP method using green solvents is essential.

Deep eutectic solvents (DESs) are low-melting-point liquid mixtures formed by mixing natural compounds consisting of hydrogen bond acceptors (HBA) and hydrogen bond donors (HBD), and are formulated according to a certain molar ratio. DESs have physical properties similar to those of ionic liquids (ILs), with similar viscosity, refractive index, high conductivity, and low surface tension (Snigur, Azooz, Zhukovetska, Guzenko, & Mortada, 2023). However, compared with ILs, DESs have the advantages of simple preparation, low toxicity, biodegradability, biocompatibility, and recyclability (Ahmadi, Azooz, Yamini, & Ramezani, 2023; Chen et al., 2022). Xu, Wang, Huang, Li, and Wen (2015) extracted bovine serum albumin (BSA) with DES (choline chloride/glycerol) and showed that 98.16% of the protein was enriched in the intermediate precipitate phase, while the conformation of the protein was not changed in the process. Rodrigues et al. (2021) used DES (betaine/propylene glycol) to extract protein from sardine processing residues and compared it with the conventional water solvent extraction method; they found that DES contains a large number of hydrophobic amino acids, such as alanine, leucine, isoleucine, and valine, which are very suitable for extracting protein from sardine processing waste. The obtained DES extract also showed excellent antioxidant and antibacterial activities. Therefore, DESs have been widely used in the extraction and preparation of plant active ingredients, including phenols, proteins, enzymes, flavonoids, and polysaccharides (Chen et al., 2022; Tebbi, Debbache-Benaida, Kadri, Kadi, & Zaidi, 2023; Wu et al., 2024; Xu et al., 2015). This study explored TPP using DES as a replacement for the traditional *t*-butanol solvent to purify tea PPO. Response surface methodology (RSM) was used to optimize the factors affecting the recovery and purification fold of tea PPO, such as the concentration of (NH_4_)_2_SO_4_, volume ratio of DES to crude extract, pH, and extraction time. Subsequently, the effects of DES recovery and recycling following purification are discussed. This study provides a new green and efficient method for the separation and purification of tea PPO using TPP technology, and provides a reference for the large-scale application of TPP to extract tea PPO.

## 2 Materials and methods

### 2.1 Material and reagents

One bud and two leaves of *Camellia sinensis* var. “Longjing No. 43” was harvested from the Jiangxi Agricultural University Tea Garden (Nanchang, Jiangxi Province, China) in May 2023. The samples were immediately frozen in liquid nitrogen and stored at −80 °C until use.

Methyl trioctyl ammonium chloride (analytically pure, 99.0%), thymol (analytically pure, 99.0%), menthol (analytically pure, 99.0%), ethylene glycol (analytically pure, ≥99.0%), glycerol (analytically pure, ≥99.5%), hexanoic acid (analytically pure, ≥98.0%), octanoic acid (analytically pure, ≥99.0%), decanoic acid (analytically pure, ≥98.5%)、dodecanoic acid (analytically pure, >98.0%), tetradecanoic acid (analytically pure, >98.0%), oleic acid (analytically pure, >99.0%), camphor (analytically pure, >97.0%), *t*-butanol (analytically pure, >98.0%), ammonium sulfate (analytically pure, >99.0%), and polyvinylpyrolidone (PVPP) were purchased from Sangon Biotech Co., Ltd. (Shanghai, China). Acrylamide, ammonium persulfate, sodium dodecyl sulfate (SDS), Tris, glycine, and Coomassie Brilliant Blue G-250 were purchased from Shanghai Suke Chemical Co., Ltd. (Shanghai, China). An Enhanced BCA Protein Assay Kit was purchased from Beyotime Institute of Biotechnology Inc. (Nanjing, China).

### 2.2 Preparation of the DESs

The temperature of the magnetic stirrer was adjusted and the DESs were prepared by heating (Tebbi et al., 2023). The HBA and HBD were mixed at an appropriate molar ratio and heated under magnetic stirring at 80 °C to form a clear and uniform mixture. The specific preparation systems and DES numbers are listed in Table 1.

**Table 1:** The details of DESs used in this study.

No.	Hydrogen bond acceptor (HBA)	Hydrogen bond donor (HBD)	Molar ratio (HBA:HBD)	Viscosity(mPa·S)	Conductivity(μs/cm)
DES-1	Methyl trioctyl ammonium chloride	Ethylene Glycol	1:2	246.667	161.133
DES-2		Glycerol	1:2	126.667	541.000
DES-3		Dodecanoic acid	1:2	366.667	12.233
DES-4		Tetradecanoic acid	1:2	195.000	10.670
DES-5	Thymol	Decanoic acid	1:1	12.333	1.241
DES-6		Octanoic acid	1:1	15.167	0.006
DES-7		Dodecanoic acid	1:1	15.000	1.208
DES-8	Menthol	Hexanoic acid	1:1	9.167	0.003
DES-9		Octanoic acid	1:1	19.167	0.004
DES-10		Oleic acid	1:1	31.833	0.178
DES-11		Dodecanoic acid	1:1	20.500	0.206
DES-12		Thymol	1:1	38.667	1.198
DES-13		Camphor	1:1	18.500	1.373

### 2.3 Characterization of the DESs

#### 2.3.1 Fourier-transform infrared (FT-IR) spectrum analysis of the DESs

FT-IR spectroscopy (Nicolet iS50 Fourier transform infrared spectrometer, Thermo Company, USA) was used to analyze the infrared spectra of the different types of DESs and compound monomers to determine conformational changes. The solid sample was mixed with potassium bromide at a ratio of 1:100 and placed in an infrared spectrometer after tableting. The liquid sample was directly dropped into the infrared spectrometer, and the reagents were scanned in the range of 400–4000 cm^−1^, with potassium bromide as the blank control.

#### 2.3.2 Viscosity of the DESs

DES viscosities were measured using a viscometer (NDJ-4 viscometer, Lichen Technology, Shanghai, China). The rotor was completely immersed in the DES at 4 °C, and under the conditions of an appropriate rotor and speed, the value was read after the rotor ran stably. Each sample was analyzed thrice to obtain average values.

#### 2.3.3 Conductivity of the DESs

DES conductivities were measured using a conductivity meter (DDS-307 A conductivity tester, Lei Magnetic Instrument Company, Shanghai, China). The electrode was completely immersed in DES. After the conductivity stabilized, the value was recorded, and each sample was analyzed thrice to obtain average values.

### 2.4 Preparation of PPO crude extract

Crude PPO was extracted according to a previously reported protocol (Teng et al., 2021), with certain modifications. Briefly, 10 g of fresh tea leaves were added to 40 mL of pre-cooled 0.1 mol/L citric acid–disodium hydrogen phosphate buffer (containing 10% glycerol (*v*/v), pH 5.6), 0.2 g of vitamin C, 1 mmol/L EDTA, and 4 g of PVPP. The mixture was homogenized for 3 min and stored at 4 °C for 2 h. Subsequently, the mixture was filtered using four layers of gauze, and the filtrate was centrifuged at 11000*g* for 20 min. The precipitate was discarded and the supernatant, which was the crude enzyme solution of PPO, was collected. All PPO experiments were performed at 4 °C without specific instructions.

### 2.5 Purification of PPO using TPP

PPO was purified via TPP, according to a previously reported protocol (Yonca, Binnur, & Arda, 2021), with certain modifications. Briefly, 20 mL of PPO crude extract was shaken and added to (NH_4_)_2_SO_4_ (40% (*w*/*v*) saturated concentration). After (NH_4_)_2_SO_4_ was completely dissolved, different types of DESs were added in a 1:1 volume ratio, mixed, and stored at 25 °C for 1 h to form a TPP system. This mixture was centrifuged at 8000*g* for 10 min at 4 °C, and the intermediate phase was concentrated into a flake protein precipitate. The upper organic and lower aqueous phases were removed, and the intermediate precipitate phase was collected and eluted with a small volume of 0.1 mol/L citrate–disodium hydrogen phosphate buffer (pH 5.6) to obtain a precipitate layer containing tea PPO. Finally, the precipitate layer was dissolved in 5 mL of 0.1 mol/L citrate–disodium hydrogen phosphate buffer (pH 5.6), PPO enzyme activity and protein concentration were determined, and the recovery and purification fold of the enzymes were calculated.

### 2.6 Assay of the PPO enzyme activity and protein content

The method described by Liao et al. (2020) was used, with slight modifications. Briefly, 10 μL of PPO enzyme solution was added to 150 μL of reaction mixture (prepared according to 0.1 mol/L citric acid–phosphate buffer: 0.1% proline: 1.0% catechol (10:2:3, v∕v∕v), pH 5.6). The blank control was replaced with a boiled enzyme solution and incubated at 37 °C for 30 min. Subsequently, 40 μL of 8 mol/L urea was added immediately to terminate the reaction. Optical density was measured at a wavelength of 420 nm using a microplate reader (Molecular Devices, LLC., USA). One unit of enzyme activity (U) was defined as the amount of enzyme that caused a change of 0.001 in the absorbance per minute.

(1) $$\text{Enzyme activity}\;\left(U\right)=\frac{\mathrm{\Delta A}}{0.001\times t}\times \frac{\text{Enzyme extract liquid volume}\;}{\text{Colorimetric volume}\times \text{Sample volume}\;}\;$$

where ΔA is the absolute value of the absorbance change over the reaction time, and t is the reaction time (min), and all volumes are in mL.

The protein content was determined using a Micro BCA Protein Assay Kit (Jiancheng Bioengineering Institute, Nanjing, China) according to the manufacturer's instructions, and BSA was used to draw a standard graph (Zhu et al., 2019). In addition, the following indicators were calculated based on enzyme activity and protein content:

(2) $$\text{Enzyme specific activity}\;\left(U/\mathrm{mg}\right)=\frac{\text{Enzyme activity}\;\left(U\right)}{\text{Protein content}\;\left(\mathrm{mg}\right)\;}\;$$

(3) $$\text{Recovery}\;\left(\%\right)=\frac{\text{Each enzyme activity}\;}{\text{Initial enzyme activity}\;}\;$$

(4) $$\text{Purification fold}=\frac{\text{Each enzyme specific activity}\;}{\text{Initial enzyme specific activity}\;}\;$$

### 2.7 Single-factor experiment

The effects of different factors on the recovery and purification fold of tea PPO were investigated. The univariate factors included the (NH_4_)_2_SO_4_ concentration (25%, 30%, 35%, 40%, 45%, 50%, and 55%), liquid volume ratio of DES-to-crude enzyme (0.1:1, 0.5:1, 1:1, 1.5:1, 2:1, 2.5:1, and 3:1), standing time (30, 45, 60, 75, 90, 105, and 120 min), and pH value (4.5, 5.0, 5.5, 6.0, 6.5, 7.0, and 7.5).

### 2.8 RSM

The primary factors affecting the extraction process were optimized using RSM (Hu et al., 2023); the (NH_4_)_2_SO_4_ concentration (*w*/*v*), DES/crude enzyme volume ratio, and pH were used as the response values. A three-level, three-factor Box-Behnken trial design was employed to predict and determine the best outcome. The (NH_4_)_2_SO_4_ concentration (40%, 45%, and 50%), B liquid volume ratio of DES-to-crude enzyme (0.1:1, 0.5:1, and 1:1), and C pH (5.0, 5.5, and 6.0) were selected as the three factors and three levels, coded as −1, 0, and + 1, and the specific level factor design is shown in Table S1. A total of 17 experimental groups were arranged, including 12 factor experiments and 5 central experiments used to estimate experimental errors.

### 2.9 SDS-PAGE and native-PAGE

To determine the purification effect and molecular weight of the enzyme, SDS-PAGE separation and stacking gel concentrations used were 12% and 5%, respectively. Briefly, 16 μL of enzyme solution and 4 μL of 5× SDS-sample buffer were added to the gel pore at 120 V. Coomassie Brilliant Blue G-250 was used as the dye. The protein molecular weight was estimated by comparing the results with a standard protein molecular weight (Solarbio Science & Technology Co., Ltd. Beijing, China) (Laemmli, 1970).

For PPO activity staining, native-PAGE was performed in a manner similar to that used for SDS-PAGE, without heating and the addition of SDS. Electrophoresis was performed on an 8% non-denaturing polyacrylamide gel. Briefly, 16 μL of the enzyme solution was added to the gel at 75 V and 4 °C. Following electrophoresis, the gel was immersed in a solution containing 0.5 mol/L sodium phosphate buffer (pH 6.8), 0.2 mol/L catechol, and 0.06% *o*-phenylenediamine (soluble in 0.01 mol/L oxalic acid). A color change was observed and pictures were obtained after 30 min (Mine, 2020).

### 2.10 Recycling and reusability of the DES

To explore the recyclability of DES in the TPP process, DES was continuously used for the TPP of tea PPO with recirculation six times under the optimal process parameters obtained in the experiment. The effects of each reused DES on the recovery and purification fold of PPO from tea were investigated.

### 2.11 Statistical analysis

SPSS Statistics17 and Origin 8.0 were used for statistical analyses. All values are expressed as the mean ± standard deviation (SD), and analysis of variance (ANOVA) was performed to ensure a 95% confidence interval. The differences between different letters in the same picture indicate statistical significance (*P* < 0.05), and all experiments were repeated three times.

## 3 Results and discussion

### 3.1 FT-IR spectra analysis of DES

The formation of DES is primarily due to the interaction between HBA and HBD. Therefore, the structures of choline chloride, glycerol, ethylene glycol, and the prepared DESs were characterized using FT-IR spectroscopy to confirm the presence of hydrogen bonds between HBA and HBD. The results are presented in Fig. S1. The infrared spectra of DES-1 (methyl trioctyl ammonium chloride/ethylene glycol), DES-5 (thymol/decanoic acid), and DES-8 (menthol/hexanoic acid) were used as examples (Fig. S1A, E, and H, respectively), and the structures of the DESs were analyzed in detail. As shown in Fig. S1A, the absorption peaks of methyl trioctyl ammonium chloride and ethylene glycol at 2918 and 2939 cm^−1^, respectively, were primarily produced by hydroxyl vibrations. In the DES-1 absorption spectrum, the ethylene glycol peak at 3298 cm^−1^ shifted to 3332 cm^−1^, indicating that methyl trioctyl ammonium chloride and ethylene glycol formed hydrogen-bonding structures (O–H…O and O–H…Cl). In addition, the strong peaks representing the CH_2_ and C—C bonds of ethylene glycol shifted from 1458 and 1031 to 1466 and 1043 cm^−1^, respectively, confirming the formation of hydrogen bonds between ethylene glycol and methyl trioctyl ammonium chloride. As shown in Fig. S1E, decanoic acid exhibited a weak hydroxyl stretching vibrational absorption band at 2916 cm^−1^ and a stretching vibrational absorption peak of C==O at 1691 cm^−1^. For DES-5, the absorption peaks of decanoic acid shifted to 2924 and 1707 cm^−1^, indicating the presence of hydrogen bonds between thymol and decanoic acid. As shown in Fig. S1H, the absorption band of caproic acid in DES-8 at 2931 cm^−1^ broadened and shifted to 2924 cm^−1^, indicating the presence of a hydrogen bond between the two components of DES-8. Similarly, the infrared spectra of the other residual DESs showed hydrogen bond formation between the receptor and donor. Changes in the absorption peaks of these groups indicate that there are interactions associated with the formation of DESs and that a large number of hydrogen bonds are formed (Chen et al., 2022; Yue, Jing, Ma, Yao, & Jia, 2012). These results confirmed that the 13 DESs selected in the experiment were successfully prepared.

### 3.2 Screening the optimal DES

**Fig. 1:**
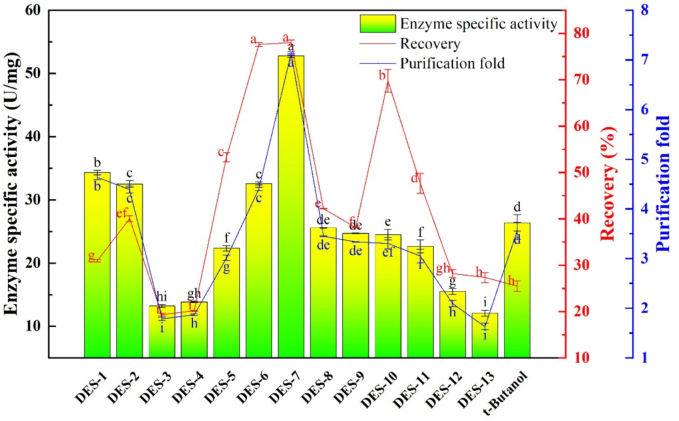
Effect of DES type on enzyme specific activity, recovery, and purification fold of tea PPO.

*t*-Butanol was used as a control to evaluate the effects of different DESs on the purification of PPO from tea. Based on the inspection indicators of PPO, namely enzyme specific activity, recovery, and purification fold, the DES species with the best purification effect on tea PPO were selected. As shown in Fig. 1, the purification effects of four types of DESs on PPO in tea were better than those of *t*-butanol. Among them, DES-7 (thymol/dodecanoic acid) exhibited the highest (*P* < 0.05) recovery and purification fold (78.04% and 7.12, respectively). In particular, the enzyme specific activity of DES-7 was up to 52.82 U/mg, which was significantly higher than that of DES-1 (34.34 U/mg; *P* < 0.05). The viscosity and conductivity of solvents are crucial properties that influence mass transfer phenomena, and as a result, their suitability for specific applications. The viscosities of the 13 DESs in the present ranged from 9.167 to 366.667 mPa·s (Table 1), and the conductivity of a DES depend on its internal hydrogen bonding network (Cao, Wu, Zhu, Dong, & Su, 2022). The effect of DES type on the extraction efficiency was extremely complex, and the physical properties of the DES, such as solubility, viscosity, conductivity, and polarity, as well as interactions between the DES and the target compound, such as hydrophobicity, hydrogen bonding, electrostatic interactions, and van der Waals forces, may affect the distribution (Wang et al., 2017). *t*-Butanol plays a role in protein flotation (Waghmare, Salve, LeBlanc, & Arya, 2016), resulting in a higher purification fold of enzyme specific activity than that associated with the use of most DESs, except DES-1, DES-2, DES-6, and DES-7. However, the PPO recovery of most DESs was higher than that of *t*-butanol (25.63%); this may be attributed to the denaturation of certain enzyme caused by the *t*-butanol. Therefore, DES-7 was selected instead of *t*-butanol for subsequent experiments to purify PPO from tea using TPP.

### 3.3 Single-factor experiment

#### 3.3.1 Ammonium sulfate concentration

**Fig. 2:**
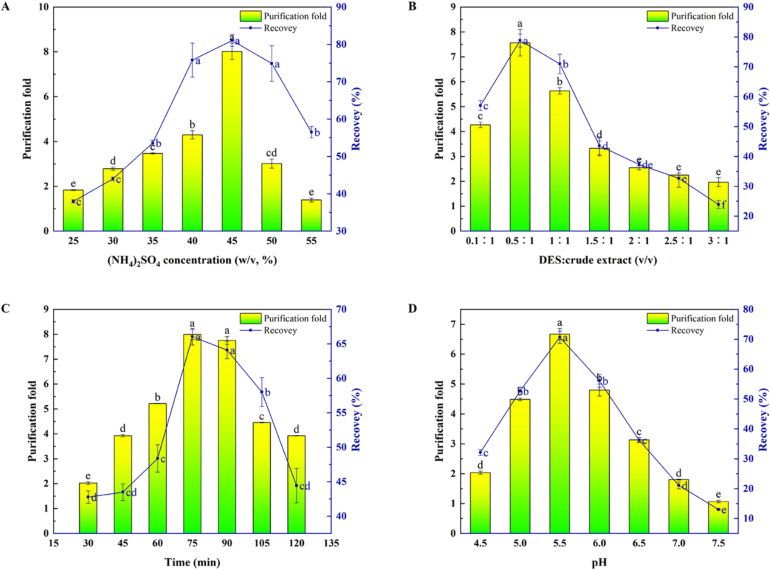
Single-factor experiments were conducted to investigate the influence of different factors on the recovery and purification fold of tea PPO. (A) Effect of (NH_4_)_2_SO_4_ concentration (*w*/*v*, %). (C) Effect of volume ratio (DES: crude extract, *v*/v). (B) Influence of standing time. (d) Influence of pH value.

The effect of (NH4) _2_SO_4_ concentration on the purification of PPO was investigated. As shown in Fig. 2A, as the concentration of (NH4)_2_SO_4_ increased, the recovery and purification fold of PPO first increased and then decreased. When the concentration of (NH_4_)_2_SO_4_ was 45% (*w*/*v*), the recovery and purification fold reached the maximum of 78.11% and 8.02, respectively. When the concentration of (NH_4_)_2_SO_4_ was low, the SO_4_^2−^ group exposed the hydrophobic group of the protein by combining with free water in the solution. With an increase in salt concentration, protein aggregates and precipitates formed owing to the hydrophobic effect. When the concentration of (NH_4_)_2_SO_4_ was too high, some of the PPO was irreversibly denatured (Chew et al., 2019). Therefore, 45% (NH_4_)_2_SO_4_ was selected for the subsequent experiments.

#### 3.3.2 Volume ratio of DES-to-crude extract

Compared with traditional solvents, pure DES exhibited a higher viscosity at room temperature, affecting mass transfer effficiency; however, adding water could reduce viscosity and increase the extraction effficiency, and the obtained proteins show a higher purity and possess better functional properties (Tebbi et al., 2023).The ratio of extraction reagent to extract is a crucial factor that determines the extraction efficiency and production cost to a significant extent. Under the same conditions ((NH_4_)_2_SO_4_ concentration of 45% and extraction time 60 min), seven different liquid-to-material ratios of DES and PPO crude enzyme solutions (0.1:1, 0.5:1, 1:1, 1.5:1, 2:1, 2.5:1, and 3:1) (*v*/v) were investigated. The results are shown in Fig. 2B, where the purification fold and recovery attained maximum values at a 0.5:1 ratio. Owing to the increased contact between the two materials, increasing the amount of solvent promoted a favorable interaction between the biomass and solvent, thus enhancing the extraction rate until equilibrium was reached. Furthermore, solvent saturation may occur at this point, thus limiting enzyme extraction (Chew et al., 2011). This phenomenon may be attributed to the insufficient synergism between DES and (NH_4_)_2_SO_4_ at low DES contents (Chen et al., 2022). When the ratio was 0.5:1, the recovery and purification fold of PPO reached maximum values. However, with increasing DES content, the recovery and purification fold decreased because the enzyme was easily denatured and inactivated when the DES content was too high. This trend was consistent with the results reported by Chen et al. (Chen et al., 2022). Therefore, a ratio of 0.5:1 between DES and crude extract (*v*/*w*) was chosen for experimentation.

#### 3.3.3 Standing time

The effectiveness of the extraction time on the TPP system under optimal parameters is shown in Fig. 2C. The purity of PPO gradually increased with time, reaching a maximum at 75 min; however, the recovery and purification fold decreased. Extraction time is an important parameter for process optimization, and extending the treatment time, which may result in the oxidation of enzymes in air and light, is not recommended (Porto & Natolino, 2018). Certain studies have demonstrated that the optimal time for enzyme extraction is approximately 30–120 min; however, this depends on the characteristics of the extracted material and the type of raw materials and type used (Panadare & Rathod, 2018; Yuzugullu Karakus, Kahveci, Acemi, & Kocak, 2020). Therefore, the experimental results obtained in this study indicated that 75 min was the optimal standing time.

#### 3.3.4 pH value

Different pH values have a significant influence on enzyme activity. Therefore, the effectiveness of the TPP system on the separation and purification of PPO under pH conditions of 4.5–7.5 were investigated. As shown in Fig. 2D, the recovery and purification fold of PPO increased with increasing pH, reaching a peak when the pH was 5.5, and then gradually decreasing when the pH was 5.5–7.5. This was because the distribution of the enzyme in the system changed when the pH was higher or lower than its isoelectric point (pI). Moreover, the enzyme tended to move to the aqueous phase because it was negatively charged when the pH of the system was higher than that of the target enzyme. When the pH of the system was lower than the pI, the enzyme accumulated during the precipitation stage because of its positive charge, easily bound to SO_4_^2−^, and precipitated the protein. In addition, in an unsuitable acid–base environment, PPO activity was reduced or even inactivated (Teng et al., 2017). A similar phenomenon was observed when the concentration of milk clotting was increased to 10 or 20 mmol/L (Hafid et al., 2020). pH 5.5 was the optimal pH value in this experiment.

### 3.4 RSM

The RSM based on Box–Behnken can analyze the regression relationship between experimental indices and factors to obtain the optimal value of independent variation. Because the extraction time had no significant effect on TPP, response surface tests of three primary influencing factors, (NH_4_)_2_SO_4_ concentration (40%, 45%, and 50% (*w*/*v*)), DES-to-crude extract ratio (0.1:1, 0.5:1, and 1:1 (*v*/v)), and pH (5.0, 5.5, and 6.0), were performed at a fixed extraction time of 75 min. The recovery and purification fold of PPO were used as indices; the results are shown in Table 2.

**Table 2:** Response surface methodology (RSM) design with the experimental result.

Run	(A) (NH_4_)_2_SO_4_ concentration (w/v, %)	(B) DES:crude extract (v/v)	(C) pH	Y1: Recovery (%)	Y2: Purification fold
1	40 (−1)	0.5:1 (0)	5.0 (−1)	45.18	5.87
2	50 (1)	0.5:1 (0)	5.0 (−1)	44.73	5.53
3	45 (0)	0.1:1 (−1)	5.0 (−1)	50.61	3.03
4	45 (0)	0.5:1 (0)	5.5 (0)	76.67	8.56
5	45 (0)	1:1 (1)	6.0 (1)	49.41	2.19
6	50 (1)	1:1 (1)	5.5 (0)	38.24	4.44
7	50 (1)	0.5:1 (0)	6.0 (1)	34.11	5.47
8	45 (0)	0.5:1 (0)	5.5 (0)	78.67	8.71
9	45 (0)	0.5:1 (0)	5.5 (0)	76.67	8.56
10	50 (1)	0.1:1 (−1)	5.5 (0)	41.75	4.87
11	45 (0)	0.1:1 (−1)	6.0 (1)	45.93	4.54
12	40 (−1)	1:1 (1)	5.5 (0)	52.72	4.53
13	45 (0)	0.5:1 (0)	5.5 (0)	77.67	8.69
14	40 (−1)	0.1:1 (−1)	5.5 (0)	57.3	4.46
15	45 (0)	1:1 (1)	5.0 (−1)	39.04	5.02
16	40 (−1)	0.5:1 (0)	6.0 (1)	61.64	4.63
17	45 (0)	0.5:1 (0)	5.5 (0)	78.67	8.73

Recovery analysis: A significance analysis was performed on the regression coefficient of the quadratic fitting model designed using the Box–Behnken test (Table 3). The *P*-value of the constant term in the model equation was <0.01, indicating that the model had a significant statistical difference. The results revealed that the effects of A, B, C, AC, BC, A^2^, B^2^, and C^2^ were extremely significant (*P* < 0.01); the AB response was not significant (*P* > 0.05); the linear and quadratic terms of A were significantly higher than those of the other factors. The R^2^ and R^2^_pred_ values of the model were 0.9989 and 0.9975, respectively, which was consistent with the R^2^_adj_ value of 0.9964. However, the lack of fit was not significant (*P* > 0.05), indicating that the model fit well and that the credibility was high (Yuan et al., 2024). The simulation equation is as follows: (See Table 4.)

**Table 3:** Analysis of Variance (ANOVA) and significance test of regression model for the recovery of tea PPO.

Source	Sum of Squares	df	Mean Square	F-value	*P*-value	Significant
Model	4094.41	9	454.93	703.28	< 0.0001	**
A- (NH_4_)_2_SO_4_ concentration (w/v, %)	420.65	1	420.65	650.27	< 0.0001	**
B- DES:crude extract (v/v)	32.72	1	32.72	50.59	0.0002	**
C-pH	16.62	1	16.62	25.69	0.0014	**
AB	0.29	1	0.29	0.44	0.5272	
AC	183.33	1	183.33	283.41	< 0.0001	**
BC	56.63	1	56.63	87.54	< 0.0001	**
A^2^	947.37	1	947.37	1464.53	< 0.0001	**
B^2^	968.64	1	968.64	1497.42	< 0.0001	**
C^2^	1112.53	1	1112.53	1719.85	< 0.0001	**
Residual	4.53	7	0.65			
*Lack of Fit*	0.53	3	0.18	0.18	0.9074	
*Pure Error*	4.00	4	1			
Cor Total	4098.94	16				
	Std. Dev. = 0.80; C.V. % = 1.44; Adeq Precision = 71.062;					
	R^2^ = 0.9989; R^2^_adj_ = 0.9975; R^2^_pred_ = 0.9964					

Level of significance: *P < 0. 05, **P < 0.01.

**Table 4:** Analysis of Variance (ANOVA) and significance test of regression model for the purification fold of tea PPO.

Source	Sum of Squares	df	Mean Square	F-value	*P*-value	Significant
Model	71.15	9	7.91	1734.84	< 0.0001	**
A-(NH_4_)_2_SO_4_ concentration (w/v, %)	0.08	1	0.08	18.44	0.0036	**
B- DES:crude extract (v/v)	0.07	1	0.07	14.22	0.007	**
C-pH	0.86	1	0.86	188.29	< 0.0001	**
*AB*	0.06	1	0.06	13.71	0.0076	**
*AC*	0.35	1	0.35	76.39	< 0.0001	**
*BC*	4.71	1	4.71	1033.30	< 0.0001	**
*A*^*2*^	6.04	1	6.04	1324.94	< 0.0001	**
*B*^*2*^	34.86	1	34.86	7650.23	< 0.0001	**
*C*^*2*^	18.17	1	18.17	3987.73	< 0.0001	**
Residual	0.03	7	0.00			
*Lack of Fit*	0.00	3	0.00	0.2	0.8937	
*Pure Error*	0.03	4	6.95E-03			
Cor Total	71.19	16				
Std. Dev. = 0.068; C.V. % = 1.17; Adeq Precision = 124.722;						
R^2^ = 0.9996; R^2^_adj_ = 0.9990; R^2^_pred_ = 0.9985						

Level of significance: *P < 0. 05, **P < 0.01.

$$Y_{1}\left(\%\right)=77.76–7.25A–2.02B+1.44C+0.27\mathrm{AB}–6.77\mathrm{AC}+3.76\mathrm{BC}–15.00A^{2}–15.17B^{2}–16.26C^{2}$$

Purification fold analysis: A significance analysis was performed on the regression coefficients of the Box–Behnken experimental design quadratic fitting model. The results are presented in Table 3. The *P* value of the constant term in the model equation was <0.01, indicating that the model had a significant statistical difference. The results showed that the effects of A, B, C, AB, AC, BC, A^2^, B^2^, and C^2^ were extremely significant (*P* < 0.01), and the linear and quadratic terms of C were significantly higher than those of the other factors. The R^2^, R^2^_adj_, and R^2^_pred_ values of the model were 0.9996, 0.9990, and 0.9985, respectively, and the model fit was extremely significant (*P* < 0.01); however, the lack of fit was not significant (*P* > 0.05), indicating that the model fit well and that the credibility was high (Yuan et al., 2024). The simulation equation is as follows:

$$Y_{2}=8.65+0.10A–0.09B–0.33C–0.13\mathrm{AB}+0.29\mathrm{AC}–1.09\mathrm{BC}–1.20A^{2}–2.88B^{2}–2.08C^{2}$$

**Fig. 3:**
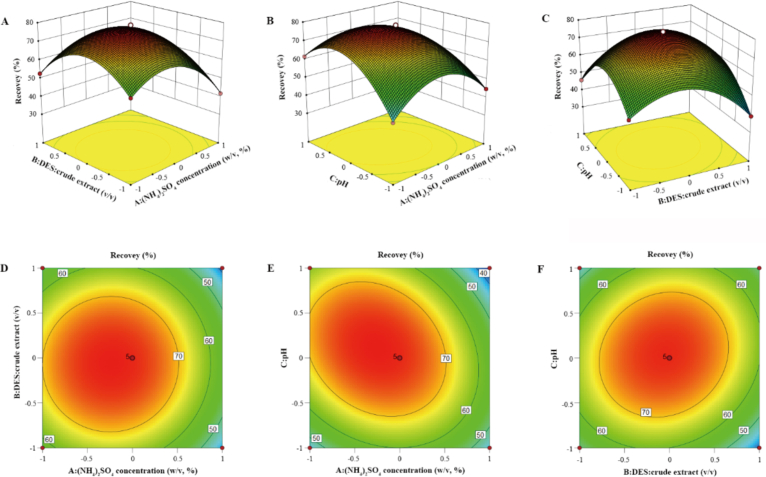
Response surface plots for tea PPO recovery. A: (NH_4_)_2_SO_4_ concentration (w/v, %); B: Volume ratio of DES to crude extract (*v*/v); and C: pH.

**Fig. 4:**
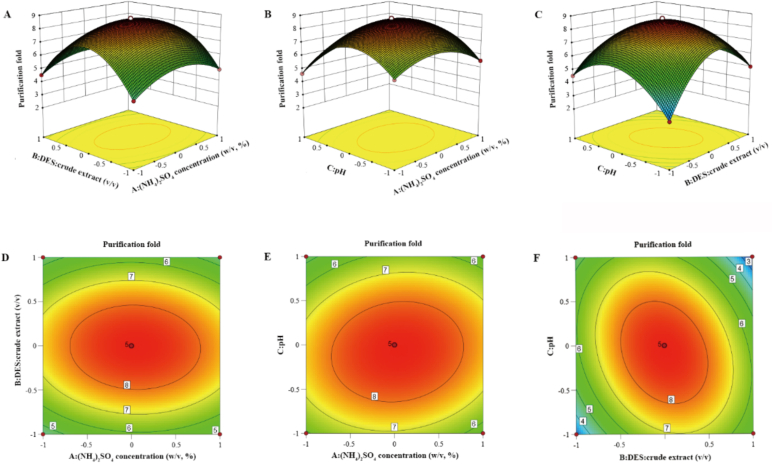
Response surface plots for the tea PPO purification fold. A: (NH_4_)_2_SO_4_ concentration (w/v, %); B: Volume ratio of DES to crude extract (v/v); and C: pH.

The interactions between the response factors on tea PPO recovery and purification fold are shown in Fig. 3, Fig. 4, respectively. The response surface slopes of the ammonium sulfate concentration, DES/crude enzyme liquid volume ratio, and pH on tea PPO recovery and purification fold were steep, the contours were oval, and the curves were dense, indicating that the interaction of various factors was significant. Simultaneously, combined with the steep surface change, the effect of ammonium sulfate concentration on the recovery of PPO was more significant, whereas the effect of pH on the purification fold of PPO was more significant, which was consistent with the ANOVA results. Finally, the response surface model was used to analyze and predict the recovery and purification fold of PPO.

### 3.5 Response surface test confirmation

Using the Box−Behnken response surface optimization test, the optimum process parameters for extracting tea PPO via the DES-based TPP method were as follows: (NH_4_)_2_SO_4_ concentration, 40.7652%; DES-to-crude extract volume ratio, 0.4933:1; and pH 5.556. Under these conditions, the PPO recovery and purification fold from fresh tea leaves were 78.717% and 8.456, respectively. To facilitate practical operation, the technical parameters were adjusted as follows: (NH_4_)_2_SO_4_ concentration, 41%; DES-to-crude extract volume ratio, 0.5: l; and pH, 5.6. Confirmation was repeated thrice under these parameters, resulting in a tea PPO recovery and purification fold of 78.44 ± 3.94% and 8.26 ± 0.41, respectively, which were close to the theoretical value and agreed with the expected model. The results showed that the technology for optimizing the TPP method of tea PPO based on the Box−Behnken response surface was reliable.

### 3.6 SDS-PAGE and native-PAGE results

**Fig. 5:**
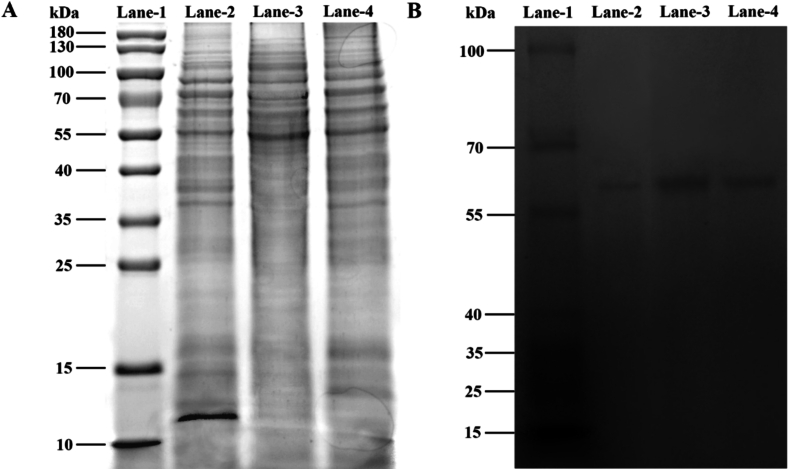
Electrophoretic patterns of tea POD after TPP. (A) SDS-PAGE and (B) native-PAGE. Lane-1 (molecular weight marker); Lane-2 (tea PPO crude extract); Lane-3 (after TPP using DES); and Lane-4 (after TPP using *t*-butanol).

To determine the effects of purification, SDS-PAGE and native-PAGE analyses were performed using the crude enzyme solution of PPO and PPO before and after TPP purification; the results are shown in Fig. 5. The SDS and mercaptoethanol use in SDS-PAGE break disulfide bonds and cause protein denaturation (peptide chain stretching), dissociating multi-subunit proteins into single subunits (Teng et al., 2021). After purification, the PPO crude enzyme solution showed relatively clear protein bands; compared to the crude extract (lane-2), the protein impurities in the channel were significantly reduced following purification with TPP (lane-3 and lane-4). In addition, the concentration of PPO after using DES-based TPP was higher than that of *t-*butanol-based TPP, which confirmed that this DES-based TPP method was more efficient than the traditional method. The native-PAGE map showed similar results. The concentration of PPO purified and enriched by DES was higher, based on the dark color of the band, and the molecular weight of PPO obtained from tea was approximately 55 kDa. This result was consistent with reports of a PPO molecular weight in tea plants (Derardja et al., 2024; Zou et al., 2024).

### 3.7 DES recovery and reusability

**Fig. 6:**
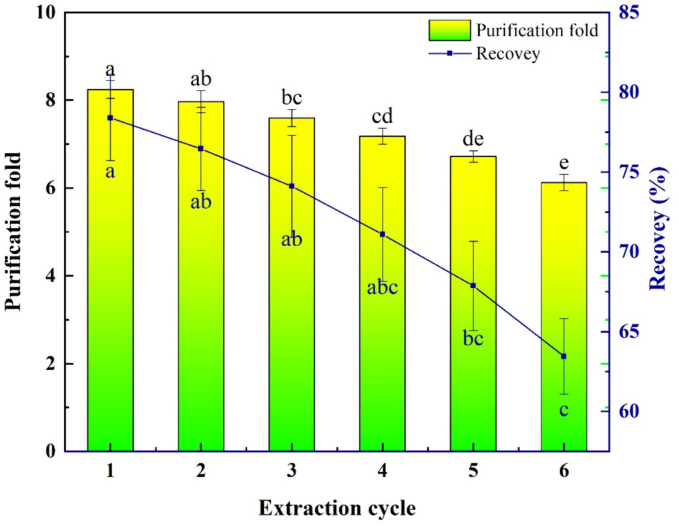
Recovery and reusability experiments of DES in TPP.

The effects of DES recovery and reusability on the purification of PPO from tea were investigated. In the process of three-phase PPO separation and purification, a certain volume of DES is added to a tea PPO crude solution containing sufficient ammonium sulfate, and after a certain period of time, the solution is separated into three phases: the lower aqueous phase, upper organic phase, and protein-rich intermediate phase between the aqueous phase and DES phase. The enzyme is assigned to the intermediate protein phase, and the impurities are assigned to the other phases, thus enabling the purification process of PPO. Therefore, after each TPP, the intermediate enzyme protein can be collected through centrifugation, while the upper DES phase is not integrated with the water phase owing to its hydrophobicity, and can be well recycled. As shown in Fig. 6, after six reusability cycles, the recovery of PPO decreased from 78.44% to 63.51%, and the purification fold decreased from 8.26 to 6.94, with decreases of 19.06% and 15.97%, respectively. It was confirmed that after six cycles of DES, the purified PPO still showed a high recovery efficiency and purification fold. Naturally, a small amount of DES solution volume is inevitably lost during each recovery, but most of it can be collected by layering, and the recovered DES can be reused into the next TPP system.

## 4 Conclusion

In this study, a DES was used instead of *t*-butanol in a TPP system to study the purification of PPO from tea. In the TPP system, a single-factor experiment and RSM were conducted. The results showed that DES-7 (thymol/dodecanoic acid) was the optimal solvent for the purification of tea PPO via TPP. Moreover, DES was superior to the traditional solvent, *t*-butanol, in terms of the purification fold and recovery in the TPP system. The optimal extraction conditions were as follows: (NH_4_)_2_SO_4_ concentration, 41%; DES:crude extract (*v*/v), 0.5:1; extraction time, 75 min; and pH, 5.6. The SDS-PAGE and native-PAGE results also revealed that DES-based TPP had a more prominent purification effect than traditional *t*-butanol-based TPP. In addition, the tests confirmed that the DES could be recovered and recycled. After repeating the tea PPO TPP method six times, DES retained its purification effects. This study implemented a DES-based TPP instead of *t*-butanol for industrial-scale tea PPO preparation. Furthermore, it can be applied as a green, efficient, and sustainable method for extracting other bioactive ingredients.

## CRediT authorship contribution statement

**Yuqin Xu:** Writing – original draft, Validation, Methodology, Investigation, Data curation. **Bin Zeng:** Writing – review & editing, Funding acquisition, Formal analysis, Conceptualization. **Shuangling Xiao:** Supervision, Methodology, Formal analysis. **Di Wang:** Project administration, Methodology, Investigation. **Yang Liu:** Writing – review & editing, Validation. **Shengchang Chen:** Validation, Methodology. **Jie Teng:** Writing – review & editing, Methodology, Funding acquisition, Conceptualization.

## Declaration of competing interest

The authors declare that they have no known competing financial interests or personal relationships that could have appeared to influence the work reported in this paper.

## Data Availability

Data will be made available on request.

## Appendix A Supplementary data

Supplementary material 1 Image 2
