# Supplementary material for: Purification of polyphenol oxidase from tea (Camellia sinensis) using three-phase partitioning with a green deep eutectic solvent
Source: Food Chem X. 2024 Aug 7;23:101720. doi: 10.1016/j.fochx.2024.101720 (PMC11369402; doi:10.1016/j.fochx.2024.101720)
Supplement: Supplementary file 1 — Supplementary material 1 [file mmc1.docx]

**Table S1**

Factor levels and variables of response surface design.

| Level | Factor | | |
| --- | --- | --- | --- |
|  | (A) (NH_4_)_2_SO_4_ concentration (w/v, %) | (B) DES:crude extract (v/v) | (C) pH |
| -1 | 40 | 0.1：1 | 5.0 |
| 0 | 45 | 0.5：1 | 5.5 |
| 1 | 50 | 1：1 | 6.0 |

**Fig. S1.** The FT-IR spectra for the DESs
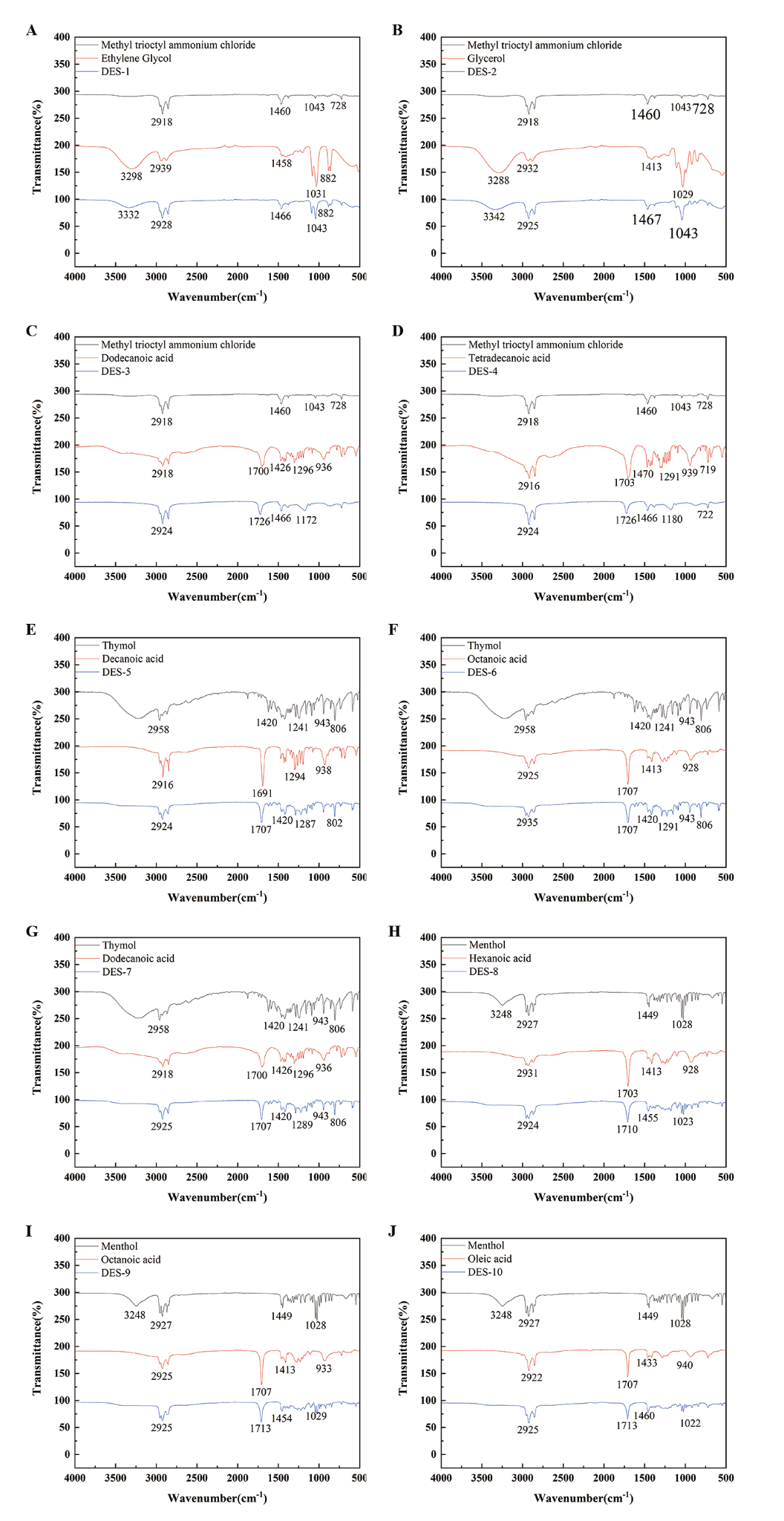
.


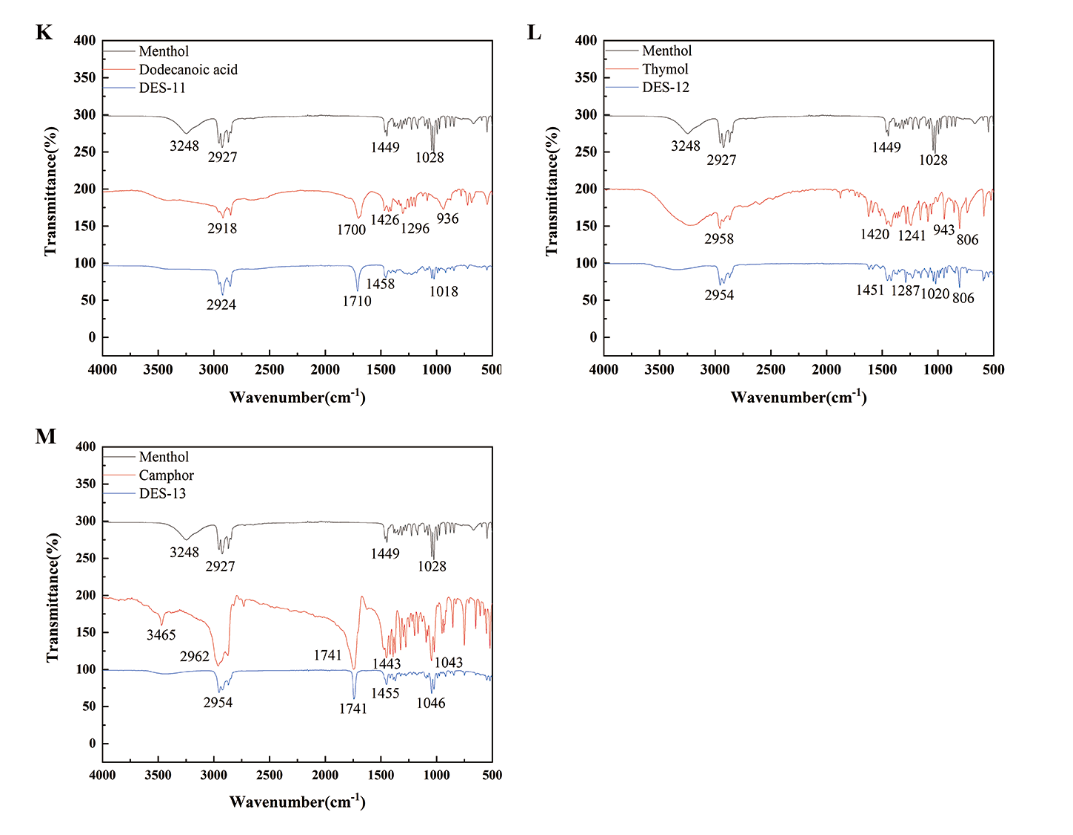
**Fig. S1.** (Continue)
